# Laser-Induced Vertical Graphene Nanosheets for Electrocatalytic Hydrogen Evolution

**DOI:** 10.1021/acsanm.4c03320

**Published:** 2024-09-25

**Authors:** Stefanos Chaitoglou, Yang Ma, Rogelio Ospina, Ghulam Farid, Jarosław Serafin, Roger Amade Rovira, Enric Bertran-Serra

**Affiliations:** † Department of Applied Physics, University of Barcelona, C/Martí i Franquès, 1, 08028 Barcelona, Catalunya, Spain; ‡ ENPHOCAMAT Group, Institute of Nanoscience and Nanotechnology (IN2UB), University of Barcelona, C/Martí i Franquès, 1, 08028 Barcelona, Catalunya, Spain; § Centro de Investigación Científica y Tecnológica en Materiales y Nanociencias (CMN), Universidad Industrial de Santander, Piedecuesta, Santander P.C. 681011, Colombia; ∥ Department of Inorganic and Organic Chemistry, University of Barcelona, C/Martí I Franquès, 1, 08028 Barcelona, Spain

**Keywords:** laser processing, graphite foil, vertical graphene nanosheets, hydrogen evolution reaction, nanostructured electrodes

## Abstract

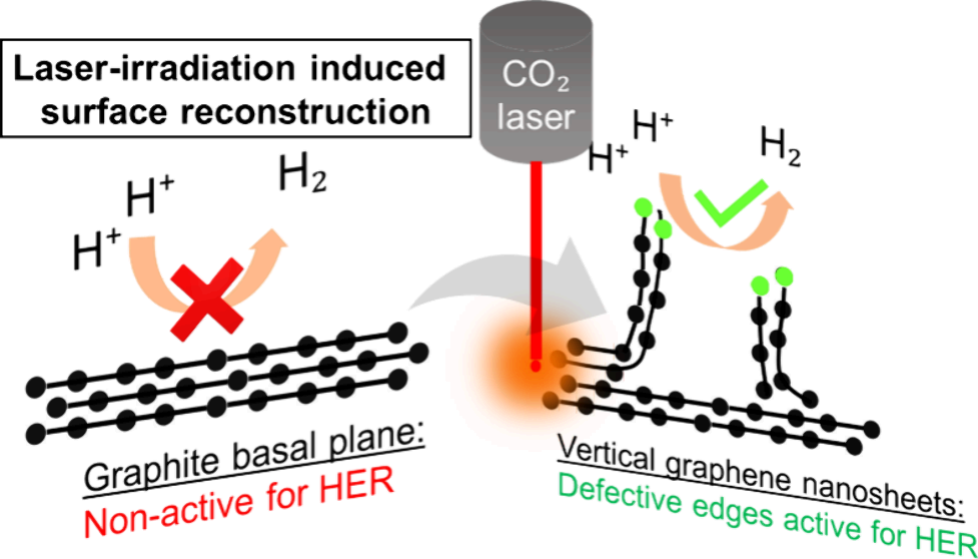

Efficient and affordable electrocatalysts are fundamental for the sustainable production of hydrogen from water electrolysis. Here, an approach for the rapid production of laser-induced vertical graphene nanosheets (LIVGNs) through the exfoliation of the graphite foil under laser irradiation is presented. The density of the formed LIVGNs is ∼3 per 100 μm^2^. On leveraging the inherent flexibility and conductivity of the graphite foil substrate, the resulting LIVGNs exhibit a 2.2-fold increase in capacitance, making them promising candidates for electrode applications. The laser-induced surface reconstruction introduces abundant sharp edges to the LIVGNs, enhancing their electrocatalytic potential for hydrogen evolution. In electrocatalytic hydrogen evolution tests in acidic media, the LIVGNs demonstrate superior performance with a remarkable decrease in the required overpotential at 10 mA cm^–2^, from −555 mV for the pristine graphite foil to −348 mV for LIVGNs. This improvement is attributed to the active sites provided by the sharp edges, facilitating hydrogen species adsorption. Furthermore, the hydrophilic behavior of LIVGNs is enhanced through the anchoring of oxygen-containing groups, promoting the rapid release of the produced hydrogen bubbles. Importantly, the modified LIVGN electrode exhibits long-term stability across a wide range of current densities during chronoamperometry tests. This research introduces a transformative strategy for the efficient preparation of vertical graphene sheets on conductive graphite foils, showcasing their potential applications in electrocatalysis and energy storage.

## Introduction

We are steadily heading toward a highly electrified society, which will permit a reduction in our dependence on fossil fuels and lower carbon emissions.^1^ Fuel cell technologies are foreseen to be a major contributor in this direction, as the storage of electricity in large quantities is still limited by current batteries.^2^ Converting electricity into a chemical fuel source, such as hydrogen, indeed offers several benefits.^3^ Consequently, the sustainable production of hydrogen is fundamental for the wide adaptation of fuel cell technologies. Toward this direction, hydrogen generation through water electrolysis has attracted a lot of attention. Unfortunately, due to the sluggish reaction kinetics of hydrogen and oxygen evolution in water splitting, catalysts are required to increase the efficiencies of these reactions.^4,5^ Noble metals, especially Pt, are considered as the most efficient electrocatalysts toward the hydrogen evolution reaction (HER), but their high cost is a bottleneck preventing large-scale application.^6^ For this reason, other metals and compounds are being tested, but corrosion resistance and efficiency are inferior.^7^ Graphene-based materials possess electronic conductivity, tunable molecular structure, and strong tolerance to both acidic and alkaline conditions, making them an ideal platform for loading cocatalysts for electrocatalytic H_2_ evolution.^8,9^ More recently, both theoretical and experimental evidence have shown that surface defect sites can serve as effective pathways to modulate the electronic structure of graphene materials and, in turn, contribute to enhancing electrocatalytic activity.^10−13^ Taking this aspect into consideration, vertical graphene nanowalls (VGNs) have emerged as a very promising electrocatalyst toward HERs,^14,15^ powered by the abundance of sharp edges that act as electrocatalytic active sites. VGNWs are conventionally synthesized by plasma-assisted chemical vapor deposition (CVD) technologies, like inductively coupled plasma CVD^16−20^ and microwave plasma-enhanced CVD.^21^ Nevertheless, CH_4_ pyrolysis requires high temperatures to overcome activation energy barriers; thus, CVD technologies for graphene synthesis require operating temperatures above 700 °C,^22−24^ and plasma power sources operate between several hundreds to thousands of Watts.^16^ An alternative method for the preparation of 3-dimensional graphene networks is based on laser irradiation of substrates, resulting in a pyrolysis process that leads to the formation of graphene nanostructures. Up to today, the most explored materials are the so-called laser-induced graphenes (LIGs), which are produced upon irradiation of polyimide (PI, with a trade name Kapton) films.^25^ The laser irradiation is performed by commercial CO_2_ lasers operating in tenths of Watts and under ambient conditions, simplifying the overall process. The produced graphene films are highly porous and conductive, making them appealing for application in energy storage systems and electrolyzers. Nevertheless, an important drawback is the fact that the PI substrate is inherently insulating, requiring additional steps for the electrical connection of the LIGs.^26^

In the present work, a novel approach for the preparation of 3-dimensional graphene networks, based on the laser irradiation of graphite foils, is presented.^27^ The laser irradiation produces a laminar exfoliation of graphite combined with surface reconstruction that results in the formation of vertically oriented graphene nanosheets. These nanosheets remain attached on the graphite foil, which is an important advantage for use in binder-free electrodes.^28^ Moreover, the graphene nanosheets possess abundant sharp edges, where defective sites are located. These defective sites are electrocatalytically active toward hydrogen evolution, which, combined with the highly conductive nature of the graphite foil, makes the resulting nanostructure a very appealing option for use as a cathode electrode in water splitting. Additionally, the highly hydrophilic surface facilitates the rapid release of produced H_2_ bubbles, preventing passivation of active sites and deterioration of the electrocatalytic efficiency.

## Experimental Part

### Materials Preparation

The flexible graphite foil was purchased from Mersen (the trade name of the foil is Papyex). The foil was cleaned with isopropanol and distilled water and allowed to dry on a hot plate. Then, the sample was loaded in a commercial CO_2_ laser engraving machine (model KH-3020 by GuangZhou Amonstar Trade Co.), operating with a power of up to 40 W. The engraving machine can be fully operated by a computer. The mirrors that direct and focus the laser beam on the substrate surface can move on both the x and y axes positioned parallel to the substrate stage. The scanning speed used was 100 mm s^–1^. Samples presented in this work were fabricated upon irradiation with the laser powers of 10 and 20 W.

### Materials Characterization

The morphology, structure, and surface chemistry of the LIVGN samples were examined using scanning electron microscopy (SEM), transmission electron microscopy (TEM), energy-dispersive X-ray spectroscopy (EDS), X-ray photoelectron spectroscopy (XPS), Raman microscope, and X-ray diffraction (XRD) measurements. All methods and apparatus are described in ref (20).

### Electrochemical Characterization

The electrochemical properties of the compounds were studied using a potentiostat/galvanostat to perform cyclic voltammetry, linear sweep voltammetry, and electrochemical impedance spectroscopy. All methods and apparatus are described in ref (8).

## Results and Discussion

**Figure 1:**
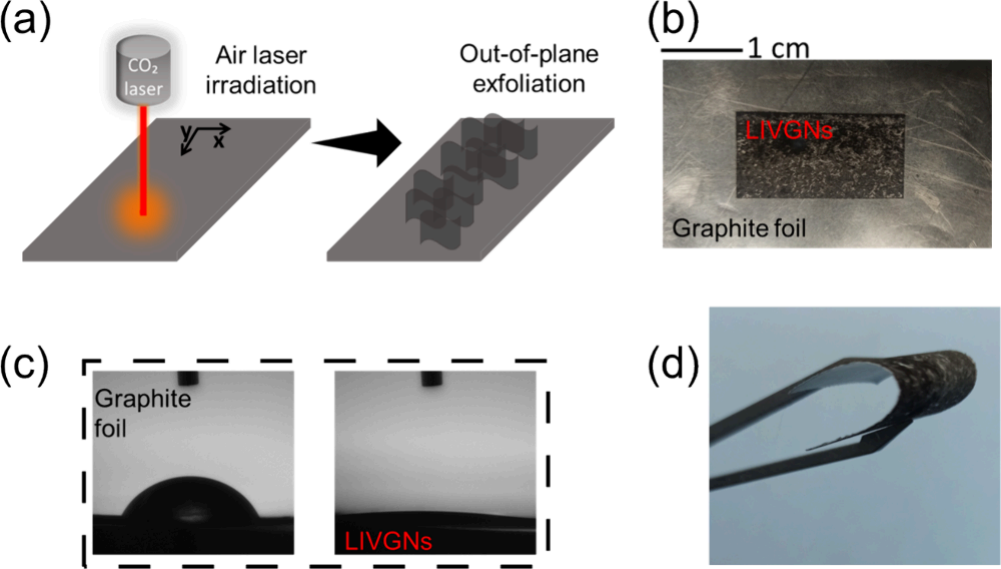
(a) Illustration of the automated CO_2_ laser irradiation process for the exfoliation and reconstruction of the LIVGNs. (b) Digital photograph of the sample upon irradiation (4 × 2 cm sample, the irradiated area marked in the center). (c) CA measurements of the pristine graphite foil (left) and the LIVGNs (right). (d) Digital photograph of the LIVGNs sample, demonstrating robust flexibility.

The processing of graphite foils and the resulting LIVGNs are depicted in Figure 1. Surface modification could be swiftly accomplished within a few minutes by scanning with a CO_2_ laser beam under ambient conditions (Figure 1a). The irradiated area appears darker in comparison to the pristine graphite foil, attributed to graphene nanosheets exhibiting increased light absorption rates, compared to those of planar graphene (Figure 1b).^29,30^ Moreover, the wettability of the surface of the LIVGNs experiences a dramatic enhancement compared to that of the graphite foil. The contact angle (CA) of a water droplet on the pristine graphite foil is ∼60° (Figure 1c, left). After irradiation, the CA reduces to less than 10° (Figure 1c, right), demonstrating the highly hydrophilic nature of the surface of the LIVGNs.^31,32^ The enhanced hydrophilicity is a result of the presence of various oxygen-containing groups attached to the surface of the LIVGNs, as shown by the XPS characterization in the respective section. An additional advantage of conducting the irradiation in air atmosphere is the elimination of the need for postgrowth plasma treatment in Ar or O_2_, a common practice applied to VGNs to grant them hydrophilic properties.^14,33^ Despite the observable surface variation, LIVGNs retain excellent mechanical flexibility (Figure 1d).

**Figure 2:**
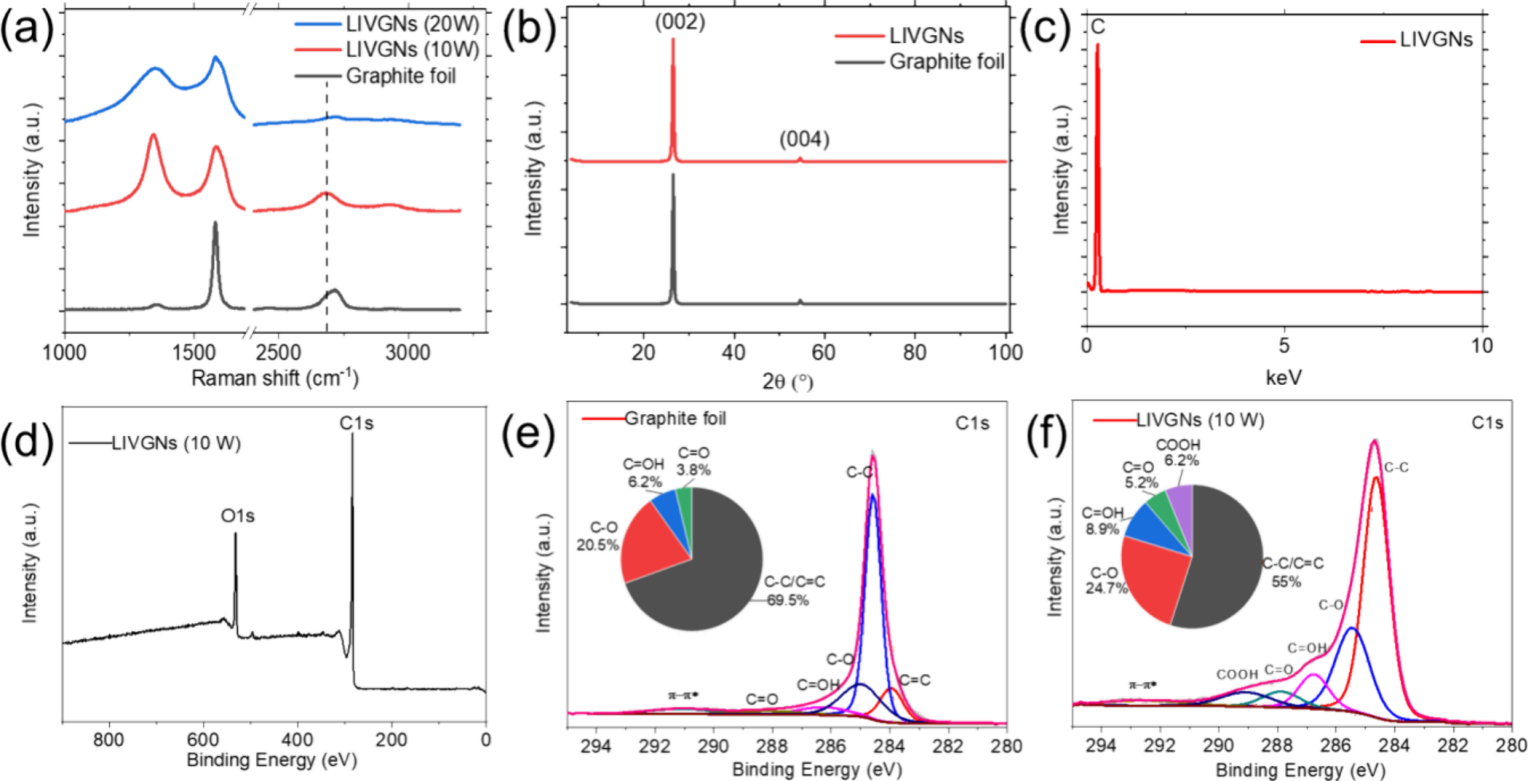
(a) Raman spectra of the pristine graphite foil (black graph), LIVGNs irradiated with a laser power of 10 W (red spectra), and LIVGNs irradiated with a laser power of 20 W (blue spectra). (b) XRD spectra of the pristine graphite foil (black graph) and LIVGNs (red spectra). (c) EDS spectrum of LIVGNs. (d) XPS spectrum of LIVGNs. (e) C 1s XPS spectrum of the graphite foil. (f) C 1s XPS spectrum of LIVGNs. Area % of components are listed for both samples in the form of inserted chart pies.

The detailed structure of LIVGNs was analyzed by Raman spectroscopy and XRD characterization. The Raman spectra are shown in Figure 2a. The spectrum of the pristine graphite foil is characterized by the presence of a sharp G band and a D-band-to-G-band peak intensity ratio (*I*_D_/*I*_G_) of 0.06 (black spectrum). For the irradiated samples, two distinct types of spectra have been collected. In the case of irradiation with a laser power of 10 W, the Raman spectrum resembles this commonly observed on VGNs. It is characterized by a D-band-to-G-band peak intensity ratio (*I*_D_/*I*_G_) of 1.30 and the rise of the 2D mode of graphene at 2682 cm^–1^. The enhanced (*I*_D_/*I*_G_) ratio reveals the presence of various structural defects on the graphene lattice, which is also promoted by the enhanced presence of edges due to the formation of vertical sheets.^34^ On the other hand, the position of the 2D peak at 2682 cm^–1^ unveils the relaxed, strain-free nature of the graphene sheets, which is compatible with the out-of-plane, vertical arrangement (in unstrained graphene, the 2D peak is positioned at 2680 cm^–1^).^35,36^ In the case of irradiation with a laser power of 20 W, the Raman spectrum resembles this commonly observed on amorphous carbon nanostructures, characterized by the widening of the D and G peaks, a decreased D-band-to-G-band peak intensity ratio (*I*_D_/*I*_G_) of 0.84, and the fading of the 2D peak.^27^ The XRD spectra are exhibited in Figure 2b. XRD patterns of both the graphite foil and LIVGNs show two typical diffraction peaks at 26.6° and 54.7°, corresponding to (002) and (004) lattices of graphite (Ref. No. 01-075-2078), respectively. The intensity of the peaks in the LIVGNs sample is slightly weakened because of the surface reconstruction. EDS characterization detects only C, proving that the fabrication process is suitable for the preparation of pure graphite electrodes free of other contaminants (Figure 2c). XPS characterization is performed to probe the surface chemistry of the nanostructure. The wide scan of the laser-irradiated sample confirms the absence of any impurities on the surface of the graphene nanosheets (Figure 2d). Comparison of the high-resolution spectra of C 1s of the pristine and laser-irradiated sample shows that the latest contains enhanced peaks at 284.6, 285.4, 286.8, 287.8, and 289.2 eV, corresponding to C–C/C=C, C–O, C=OH, C=O, and COOH groups, respectively (Figure 2e,f).^27,37^ A comparison of the area % of components between the two samples, in the form of inserted chart pies, shows the enhanced presence of the oxygen-containing groups in the laser-irradiated sample. The oxygen-containing functional groups introduced into carbon materials are generally hydrophilic groups, such as the carboxyl and hydroxyl groups. In these groups, oxygen has strong polarity and can be associated with the hydrogen bond of water molecules by the dipolar force, so it has relatively strong activity and shows strong wettability.^38^ These results demonstrate that laser irradiation of the graphite foil in air leads to the formation of numerous oxygen-containing functional groups that are firmly attached to the surface of the resulting LIVGNs.

**Figure 3:**
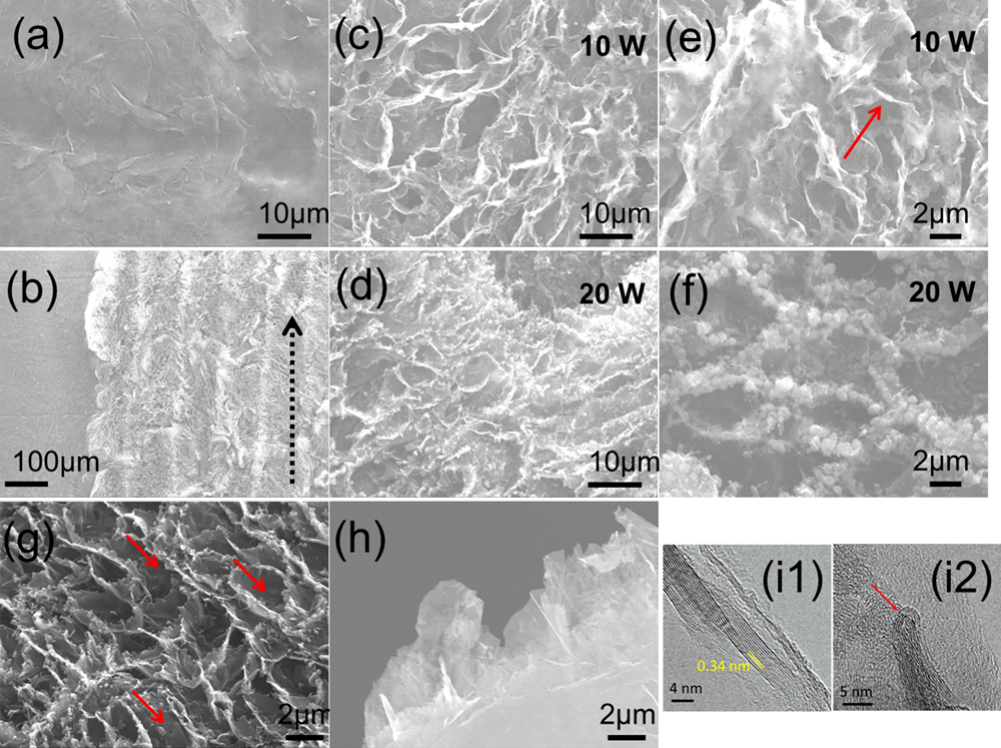
Microscopic characterization. Top-view SEM images of: (a) pristine graphite foil, (b) reconstructed surface upon irradiation (black arrow indicates the scanning direction), (c,e) LIVGNs irradiated with a laser power of 10 W (red arrow indicates the sharp edges), and (d,f) LIVGNs irradiated with a laser power of 20 W. SEM images of the LIVGNs (g) tilted in 45° (red arrows mark the nanosheet orientation) and (h) tilted in 90°. (i1,2) HRTEM images exposing the lattice fringes and sharp edges of LIVGNs (red arrow indicates the sharp edges, Figure i2).

SEM characterization provides insights regarding the morphological features of LIVGNs (Figure 3). The pristine graphite foil exhibits a smooth surface composed of densely stacked graphene layers (Figure 3a). Laser irradiation patterning is visible on the reconstructed area, revealing various grooves with a width of ∼100 μm each. These grooves result from continuous linear laser scanning, with their width depending on the laser beam′s diameter (Figure 3b). Higher magnification images reveal the nanostructured morphology of the LIVGNs. Samples irradiated with both 10 and 20 W of laser power show a similar morphology, characterized by the vertical arrangement of the graphene nanosheets upon irradiation (Figure 3c,d). The density of the formed LIVGNs is ∼3 per 100 μm^2^. The distance between distinct nanosheets is ∼2 μm. Their length is ∼2–3 μm. This alternative morphology arises as a result of the graphite surface reconstruction induced by high-power input,^27,39^ leading to a strong degassing process located on the surface of the graphite paper. The observed vertical alignment suggests the creation of laser-induced defects in the graphene lattice, which provoke a permanent strain-induced out-of-plane bending of the nanosheets.^40^ By careful observation of the nanosheet-exposed terminations, two distinct morphologies emerge. For samples irradiated with a laser power of 20 W, micrometric particles are deposited on the nanosheet terminations (Figure 3f). These particles originate from a process including graphite melting, evaporation, and subsequent redeposition^41^ and are highly amorphous, as revealed by Raman spectroscopy characterization. Samples irradiated with a laser power of 10 W present sharp edges, nonaltered by the redeposited material (Figure 3e). The Raman fingerprints of these samples resemble those of VGNWs. The vertical arrangement and sharp edges of the nanosheets can be further appreciated in Figure 3g, where the sample is tilted in 45°, and in Figure 3h, where the side view of LIVGNs is presented. TEM characterization provides insights into the structural characteristics of the LIVGNs. High-resolution images of the sample exposed to a laser power of 10 W are presented in Figure 3i1,2. Figure 3i1 reveals the multilayer nature of the graphene nanosheets, with a characteristic *d*-spacing value of 0.338 nm.^42^ Moreover, the sharp edges of the nanosheets can be distinguished (Figure 3i2). The thickness of the nanosheets is ∼4 nm, corresponding to graphene stacks consisting of ∼12 atomic layers.

**Figure 4:**
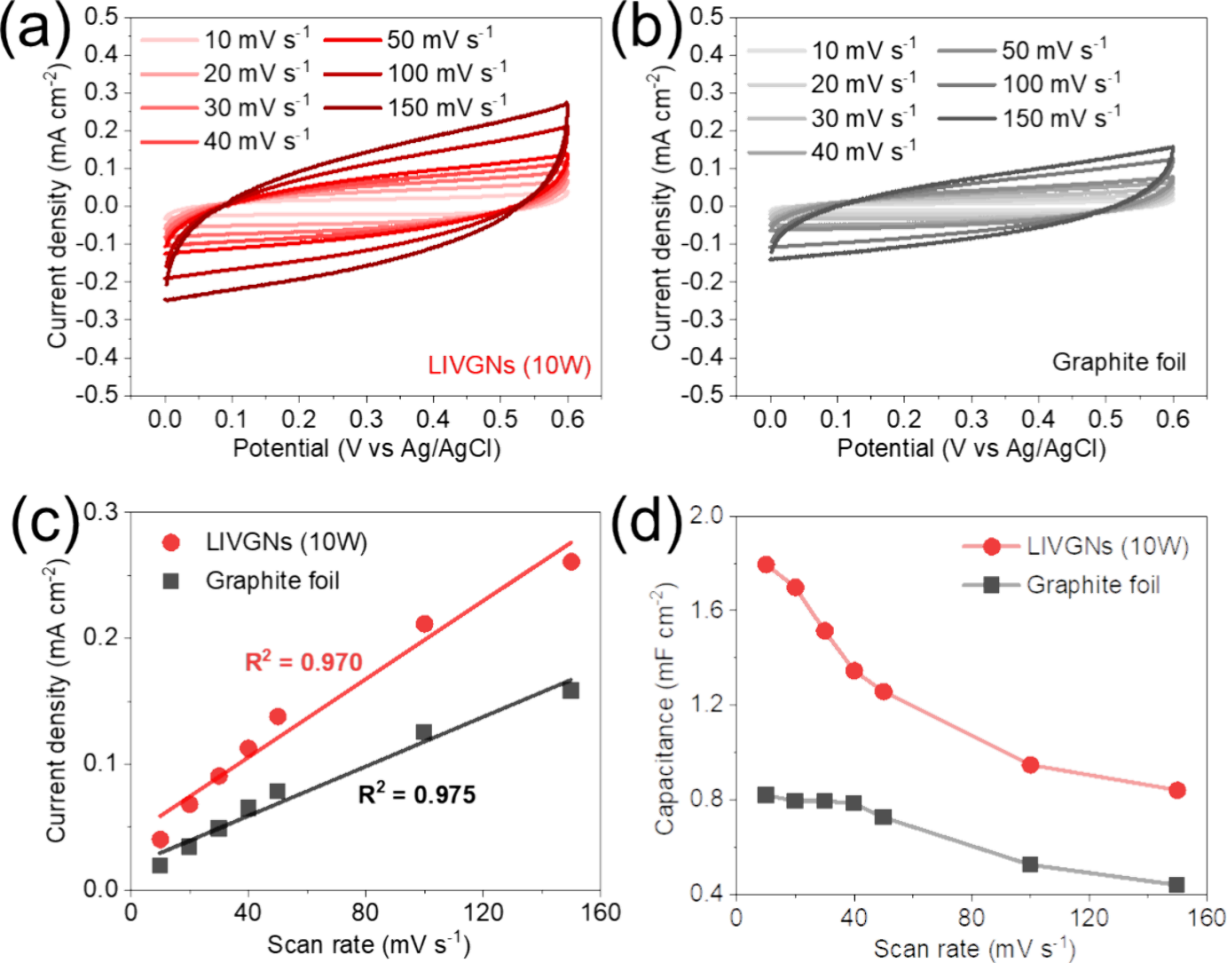
CV curves for (a) LIVGNs (10 W) and (b) graphite foil electrodes at various scan rates, (c) plot of current density versus scan rate for the LIVGNs (10 W) and graphite foil electrodes, and (d) capacitance plots for the LIVGNs (10 W) and graphite foil electrodes derived from the CV curves.

To demonstrate the enhanced electrochemical properties of the LIVGNs electrode, the CV curves for LIVGNs (10W) (Figure 4a) demonstrate a pronounced capacitive behavior that is maintained across a wide range of scan rates from 10 to 150 mV s^–1^. This is evidenced by the progressive increase in the current density with the scan rate, indicative of excellent charge storage and rapid electrochemical kinetics. In contrast, the graphite foil electrode (Figure 4b) exhibits lower current densities at the corresponding scan rates, suggesting a comparatively lower electrochemical activity. In Figure 4c, the linear regression of the current density versus the scan rate reveals high correlation coefficients (*R*^2^ values above 0.970), affirming the capacitive nature of the electrodes.^43^ The steeper slope for LIVGNs indicates fast electronic and ionic transport, which improves the energy storing and rate capability. Figure 4d illustrates a comparison of the specific capacitances calculated from the CV curves for all tested samples at different scan rates. It becomes evident that the LIVGNs (10 W) electrode has the highest capacitance, reaching 1.795 mF cm^–2^ at a scan rate of 10 mV s^–1^, which is 2.2 times higher than the capacitance of the graphite foil (0.819 mF cm^–2^). It can be seen that the capacitance decreases with an increasing scan rate. This is because at lower scan rates, the electrolyte ions have more time to diffuse throughout the electrode material. The results indicate that LIVGNs (10 W) exhibit superior capacitive performance compared to the graphite foil, with higher current densities and high-rate capability. In fact, the enhanced electrochemical performance of the LIVGNs electrode is attributed to its significantly larger specific surface area because of its unique microstructure architecture compared to the graphite foil, which facilitates efficient ion transport and provides abundant active sites for charge storage.

**Figure 5:**
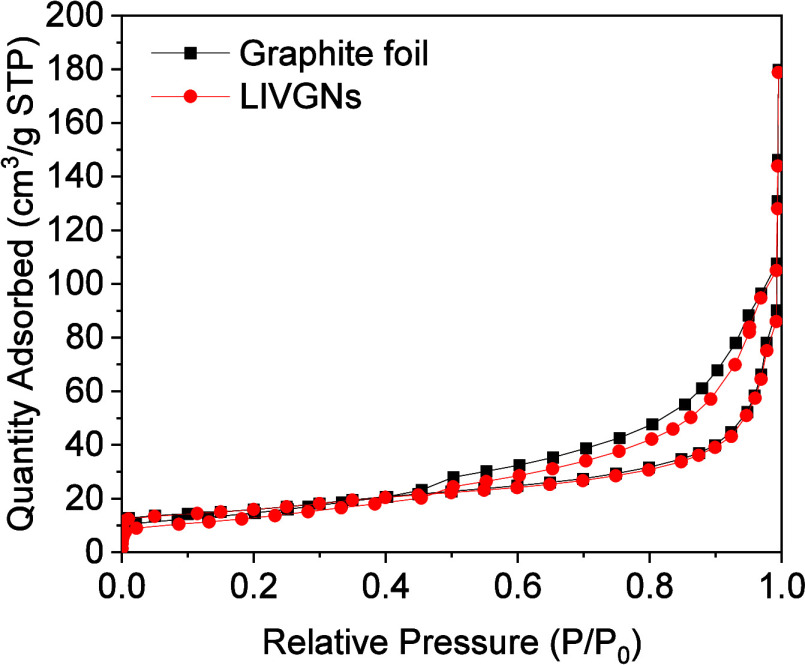
N_2_ adsorption–desorption isotherms at 77 K for the graphite foil (black points) and LIVGNs (red points).

Analysis of the nitrogen adsorption–desorption data at 77 K for the pristine graphite foil and LIVGNs is presented in Figure 5. The type IV isotherm observed for both materials is the characteristic of porous materials, particularly those with mesopores.^44^ In the initial phase of the relative pressure increase (*P*/*P*_0_ < 0.1), the amount of adsorbed nitrogen rises gradually. This initial increase indicates monomolecular adsorption, primarily occurring on the surface of available adsorption sites. In the subsequent stage, particularly at a relative pressure of *P*/*P*_0_ ≈ 0.9, there is a marked acceleration in the increase in the volume of adsorbed nitrogen. This phenomenon results from capillary condensation in the mesopores, where the porous structures begin to be more filled with nitrogen. As the relative pressure increases, the available adsorption sites become saturated, leading to an accelerated adsorption rate due to the occupation of new sites in the material’s pores. Detailed analysis of the textural parameters reveals significant features of the pore structure of both materials. For the pristine graphite foil, the specific surface area is 39.93 m^2^/g, the total pore volume is 0.28 cm^3^/g, and the average pore width is 19.81 nm. These values indicate a relatively uniform porous structure with a moderate pore volume and a relatively large average pore width. In the case of LIVGNs, the specific surface area is 55.72 m^2^/g, the total pore volume is 0.28 cm^3^/g, and the average pore width is 19.87 nm. LIVGNs have a slightly larger specific surface area than the pristine graphite foil, and the average pore width is marginally wider by 0.06 nm. This subtle increase in the pore width could be attributed to the laser processing applied to LIVGNs, potentially leading to structural modifications that result in slightly wider pores. The hysteresis loop observed in the adsorption–desorption isotherms provides additional information about the pore structure. For both samples, the presence of type H4 hysteresis loops can be observed, indicative of a material with a diverse pore size distribution encompassing both mesopores and macropores. The type H4 hysteresis is typically observed in materials with such varied pore structures, reflecting a broad range of pore sizes that include mesopores (2–50 nm) and macropores (>50 nm).^44^ For the pristine graphite foil, the hysteresis loop exhibits a well-defined type H4 pattern, suggesting a porous network with significant capillary condensation. The clear loop is the characteristic of materials with a broad pore size distribution, which facilitates the adsorption and desorption processes of nitrogen. This well-defined hysteresis indicates that the graphite foil has a substantial presence of mesopores and macropores, which contribute to its adsorption properties. In the case of LIVGNs, the hysteresis loop also corresponds to type H4, but with a less pronounced profile compared to that of the pristine graphite foil. This reduced clarity in the hysteresis loop for LIVGNs suggests that while it retains a broad pore size distribution, the laser-induced modifications have introduced structural changes in the porous network. These changes may include variations in pore connectivity or the formation of additional surface modifications, leading to a less distinct but still recognizable Type H4 hysteresis loop.

In the context of electrocatalytic hydrogen evolution, materials with an optimal pore structure that facilitate effective transport and molecular interactions are crucial. The pristine graphite foil, with its uniform pore structure, may provide stable and repeatable catalytic properties, which are critical for applications requiring consistent performance. The uniformity of the pristine graphite foil’s porosity may contribute to stable results in HERs but could limit its effectiveness in more complex electrocatalytic processes. On the other hand, LIVGNs, with its slightly larger surface area and more varied pore structure, may offer enhanced catalytic properties for HERs. The increased average pore width and diversity in the porous structure could create additional active sites and improve the availability of active sites for reagent adsorption. Consequently, LIVGNs may exhibit superior efficiency in electrocatalytic hydrogen evolution, making them advantageous for more demanding electrocatalytic applications.

**Figure 6:**
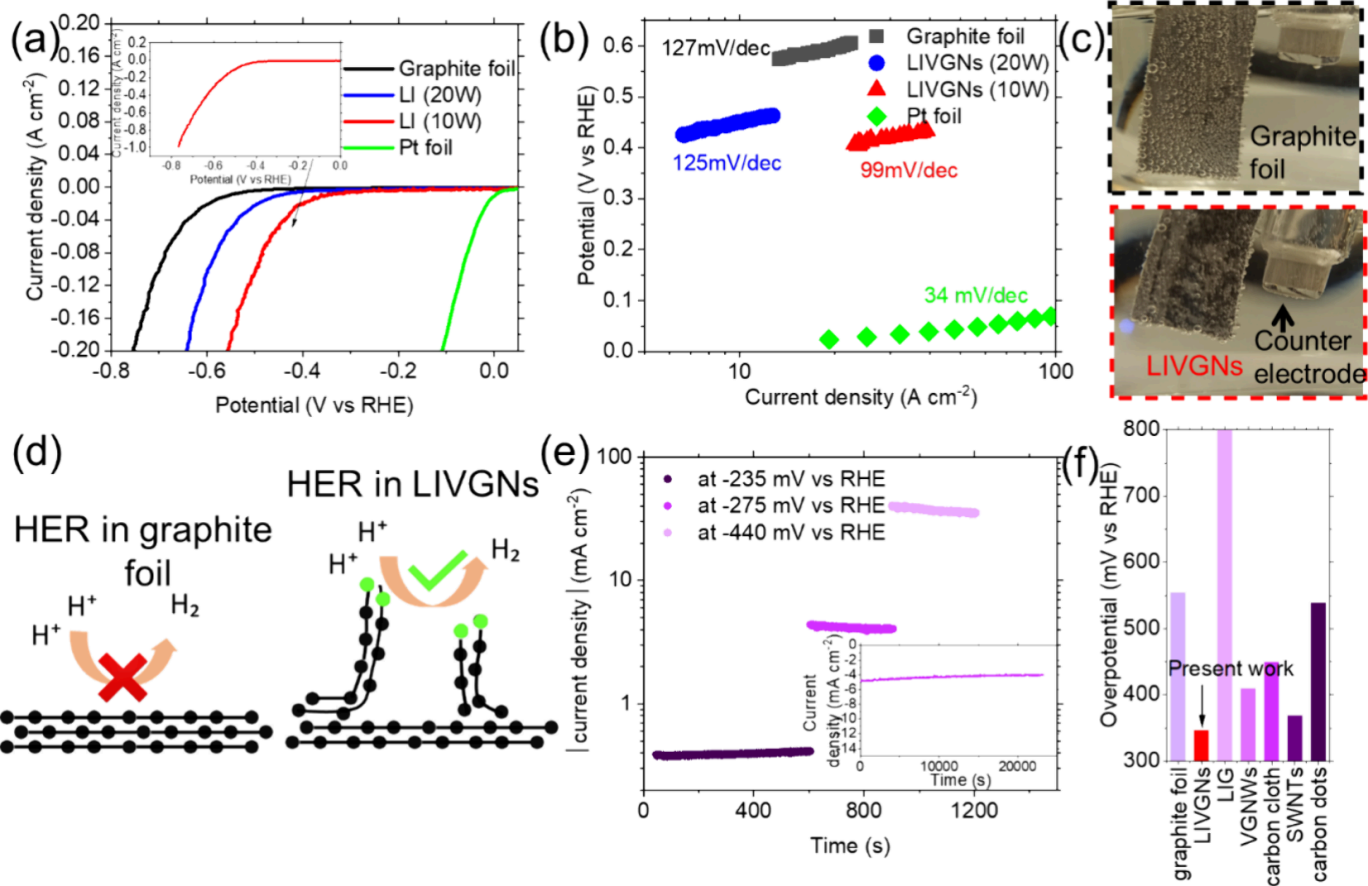
(a) LSV curves of the pristine graphite foil (black curve), Pt foil (green curve), the LIVGNs irradiated with a laser power of 20 W (blue curve), and the LIVGNs irradiated with a laser power of 10 W (red curve). Inset of (a) shows the wide scan LSV curve of LIVGNs irradiated with a laser power of 10 W. (b) Tafel slopes of the pristine graphite foil (black curve), Pt foil (green curve), the LIVGNs irradiated with a laser power of 20 W (blue curve), and the LIVGNs irradiated with a laser power of 10 W (red curve). (c) Digital photographs of the graphite foil (top photograph) and LIVGNs (bottom photograph) during hydrogen evolution at a steady overpotential of −590 mV. (d) Illustration comparing the HER mechanism between the graphite foil and LIVGNs. (e) Chronoamperometry test of the LIVGNs electrode at varying overpotential values. (f) Histogram with comparison of the required overpotential values for generation of 10 mA cm^–2^ for various carbon-based nanostructured materials.

The pristine graphite foil and nanostructured LIVGNs were tested as working electrodes to study their electrocatalytic properties. The electrocatalytic properties of the HER in an acidic medium were evaluated by LSV. The results are presented in Figure 6a. As expected, the Pt foil electrocatalyst still showed the best performance, since it requires 0 onset potential and approximately −0.02 V to generate 10 mA/cm^2^ (green curve). The pristine graphite foil shows an onset potential of −330 mV and an overpotential of −555 mV for the generation of 10 mA/cm^2^ (black curve). The LIVGNs sample irradiated with a laser of 20 W power shows an onset potential of −240 mV and an overpotential of −450 mV for the generation of 10 mA/cm^2^ (blue curve). The enhanced performance is attributed to the increase in the available active surface area, as a result of the vertical arrangement of the graphene nanosheets, as well as the increase in the density of structural defects, as indicated by the respective Raman fingerprints, which are favored to serve as catalytically active sites for the hydrogen evolution. The LIVGNs sample irradiated with a laser of 10 W power shows an onset potential of −235 mV and an overpotential of −348 mV for the generation of 10 mA/cm^2^ (red curve). The wide scan LSV curve of this sample is presented in the inset of Figure 6a. Approximately 1 A cm^–2^ is generated at −800 mV, confirming that the present electrode can sustain very high current densities, which is crucial for high production volumes.^45^ Cyclic voltammetry in the HER region shows complete reversibility of the process, pointing toward the conclusion that the recorded current is attributed to ion diffusion (Figure S1). Moreover, LSV measurements performed with the graphite foil used as a counter electrode showed an almost identical electrochemical performance (Figure S2). The catalytic activity of the LIVGNs has been recorded in alkaline conditions to compare with the one recorded in acid conditions (Figure S3). A ∼ 200 mV increase in the onset potential is observed, demonstrating that hydrogen evolution occurring in alkaline conditions, although viable, is less efficient. XPS characterization of the electrochemically tested LIVGNs sample reveals the rise of an additional O 1s component, in lower energy than the main peak centered at 530.6 eV (Figure S4a). The main peak is attributed mainly to C=O bonds, inherent in the LIVGNs structure, while the more electronegative peak appears due to the reaction of oxygen with potassium present in the electrolyte, as confirmed by the wide scan measurement (Figure S4b). The superior performance is attributed to the enhanced presence of sharp edges on the terminations of the LIVGNs, enriching the density of the available active sites. This finding is supported by density functional theory calculations performed elsewhere, which have shown that these sharp-edge sites are more active toward proton reduction and have higher charge density, which favors the process of hydrogen evolution. The Gibbs free energy of H* adsorption in the basal plane of graphene is 1.15 eV, indicating that hydrogen cannot efficiently adsorb onto the surface. On the other hand, the Gibbs free energy of H* adsorption on sites located on sharp edges is −0.38 eV, demonstrating that these are more active toward proton reduction.^13^ This is a consequence of the enhanced presence of oxygen-containing functional groups, which favor association with protons, thus initiating hydrogen evolution.^46^ Illustration with a comparison of the HER mechanism occurring in the pristine graphite foil and LIVGNs is provided in Figure 6d, to showcase the efficiency of the latest due to the formation of electrocatalytically active sharp edges. Respected Tafel slopes are presented in Figure 6b. Tafel slope values provide information regarding the reaction rates and identify the rate-limiting step during hydrogen evolution. They are used to report the required minimum overpotentials to obtain a current density increase by 1 order of magnitude. The pristine graphite foil shows a Tafel slope of 127 mV per decade, which indicates that the H^+^ adsorption step, or the Volmer step, is the rate-determining step. The LIVGNs irradiated by the laser with a power of 20 W show a Tafel slope of 125 mV per decade, indicating slightly faster reaction kinetics. Nevertheless, similar to the case of pristine graphite foil, the Volmer step is the rate-determining step. Finally, the LIVGNs irradiated by laser with a power of 10 W show a Tafel slope of 99 mV per decade, indicating much faster reaction kinetics, governed by the fact that a mixed Volmer–Heyrovsky mechanism determines the reaction rate.^47^ The Pt foil shows a slope of 34 mV per decade. To obtain a more in-depth understanding of the enhanced performance of LIVGNs, two digital photographs of LIVGNs and the pristine graphite sample are compared (Figure 6c). Both photographs were taken while applying a steady overpotential of −590 mV. In the pristine graphite foil, the produced H_2_ gas forms enhanced bubbles that remain attached to the sample surface (top photograph). This is a result of the hydrophobic nature of the graphite foil, which prohibits the fast release of the H_2_ bubbles, resulting in partial passivation of the bubble-covered surface and deterioration of the electrocatalytic efficiency.^48^ On the other hand, the hydrophilic nature of the LIVGNs, the result of the presence of sharp edges and oxygen-containing groups attached on the surface, drives forward the rapid release of H_2_ bubbles (bottom photograph), enabling a more efficient electrocatalytic process.^49^ The endurance of the LIVGNs toward the HER is further explored by chronoamperometry tests performed under varying steady overpotential values (Figure 6e). Applied overpotentials were selected with the aim of generating current density values of varying orders of magnitude, targeting to explore the electrochemical stability of the electrode in a wide operational range. It is observed that for current densities of 0.4, 4, and 40 mA cm^–2^, the electrode demonstrates remarkable stability. Remarkable stability for 6 and 20 h is recorded for the case of 4 mA cm^–2^ (Figure 6e, inset) and ∼6 mA cm^–2^ (Figure S5) in acid and alkaline conditions, respectively. A histogram comparing the overpotential values of various carbon-based nanomaterials required for the generation of 10 mA cm^–2^ is presented in Figure 6f. The present LIVGNs are placed as the most efficient option, requiring the lowest overpotential, when compared with LIG,^2^ graphite foil (present work), VGNWs,^14^ single-call carbon nanotubes,^50^ carbon dots,^51^ and carbon cloth.^52^ In addition, the HER performance is comparable to that of transition metal-based compounds reported previously in the literature.^53−55^

## Conclusions

The current investigation unveils a straightforward and innovative process for crafting vertical graphene nanosheets through the CO_2_ laser irradiation of the graphite foil, conducted under ambient conditions. The applied thermal budget induces a laminar exfoliation of graphite, coupled with surface reconstruction, culminating in the formation of graphene nanosheets arranged vertically. Raman spectra analysis discerns that a laser operating at 10 W yields graphene nanosheets, whereas at 20 W, the irradiation results in the formation of amorphous-like nanosheets. XPS characterization brings to light the emergence of oxygen-rich groups, pivotal in augmenting the surface’s hydrophilic behavior. Electrochemical characterization of the laser-induced vertical graphene nanosheets (LIVGNs) demonstrates a striking 2.2-fold surge in capacitance compared to the pristine graphite foil. Importantly, these LIVGNs exhibit noteworthy electrocatalytic processes in hydrogen evolution. The heightened electrocatalytic performance can be ascribed to the abundant structural defects and sharp edges, both serving as active sites for H* adsorption. Notably, the LIVGNs electrode achieves a current density of 10 mA cm^–2^ at an overpotential of −348 mV, marking a substantial reduction of 307 mV in comparison with the pristine graphite foil. Furthermore, the LIVGNs electrode showcases exceptional stability across a spectrum of current densities, ranging from 0.4 to 40 mA. This study offers novel perspectives on the generation of two-dimensional nanosheets with a vertical orientation through laser-induced exfoliation. The methodologies presented can be applied to the broader family of two-dimensional layered materials and boost their applicability in catalysis and energy storage.

## Supporting Information Available

CV measurement of the LIVGN electrode, LSV measurement, comparison of LSV curves in acid and alkaline conditions, XPS graph of the LVGN electrode, and chronoamperometry test of the catalyst in alkaline conditions (PDF)
