# Supplementary material for: Laser-Induced Vertical Graphene Nanosheets for Electrocatalytic Hydrogen Evolution
Source: ACS Appl Nano Mater. 2024 Sep 25;7(19):22631–9. doi: 10.1021/acsanm.4c03320 (PMC11480902; doi:10.1021/acsanm.4c03320)
Supplement: Supplementary file 1 — an4c03320_si_001.pdf [file an4c03320_si_001.pdf]

Supporting Information: Laser-Induced Vertical Graphene Nanosheets for Electrocatalytic Hydrogen Evolution.

Stefanos Chaitoglou[1,2,*], Yang Ma[1,2], Rogelio Ospina[1,2,3], Ghulam Farid[1,2], Jarosław Serafin[1,2,4], Roger Amade Rovira[1,2], Enric Bertran-Serra[1,2]

1. Department of Applied Physics, University of Barcelona, C/Martí i Franquès, 1, 08028 Barcelona, Catalunya, Spain
2. ENPHOCAMAT Group, Institute of Nanoscience and Nanotechnology (IN2UB), University of Barcelona, C/Martí i Franquès, 1, 08028 Barcelona, Catalunya, Spain
3. Centro de Investigación Científica y Tecnológica en Materiales y Nanociencias (CMN), Universidad Industrial de Santander, Piedecuesta, Santander P.C. 681011, Colombia
4. Department of Inorganic and Organic Chemistry, University of Barcelona, C/Martí I Franquès, 1, 08028 Barcelona, Spain

Corresponding author e-mail address: stefanoschaitoglou@ub.edu (S. Chaitoglou)

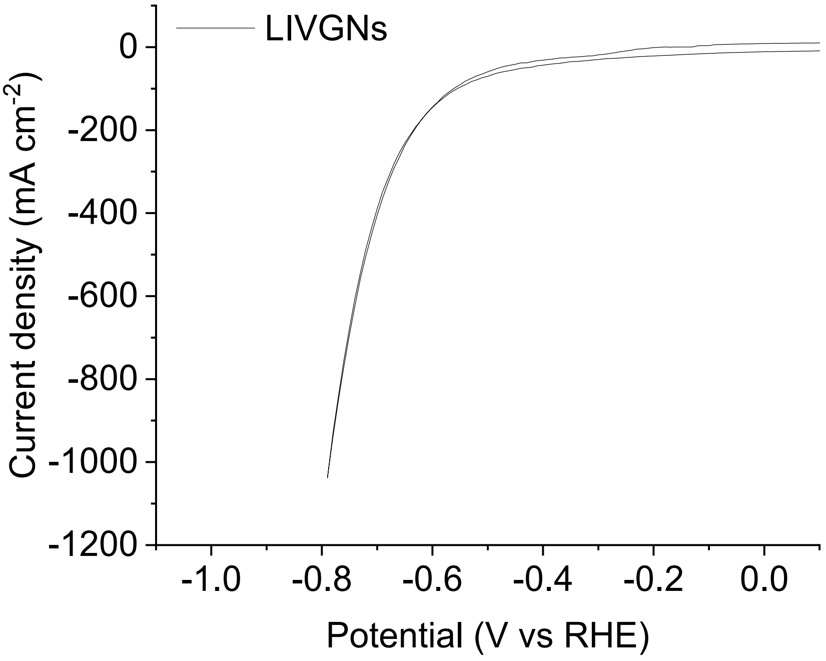

Figure S1: CV measurement of the LIVGNs electrode on a scan rate of 10 mV sec^{-1}.

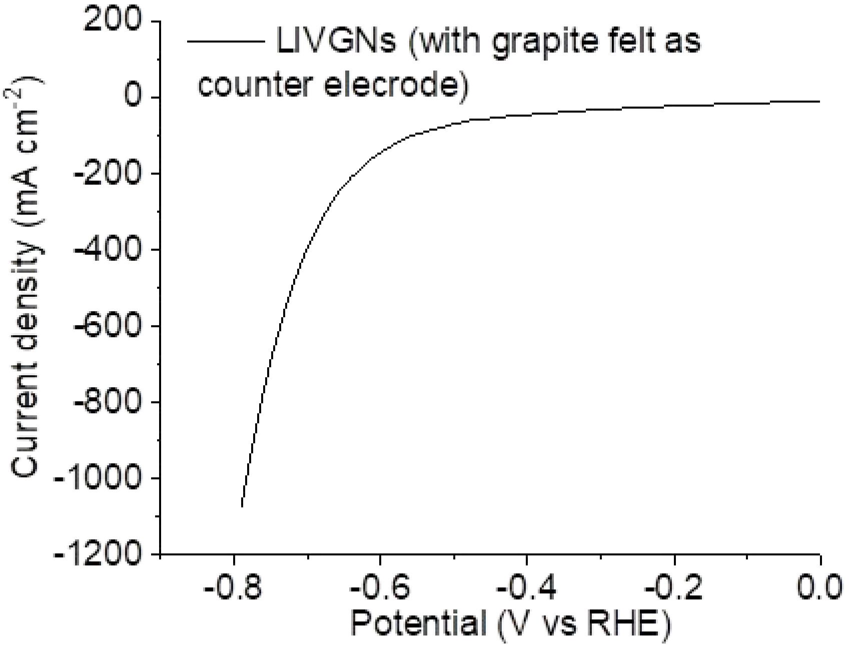

Figure S2: LSV measurement where LIVGNs on graphite paper is used as working electrode and a graphite felt is used as counter electrode.

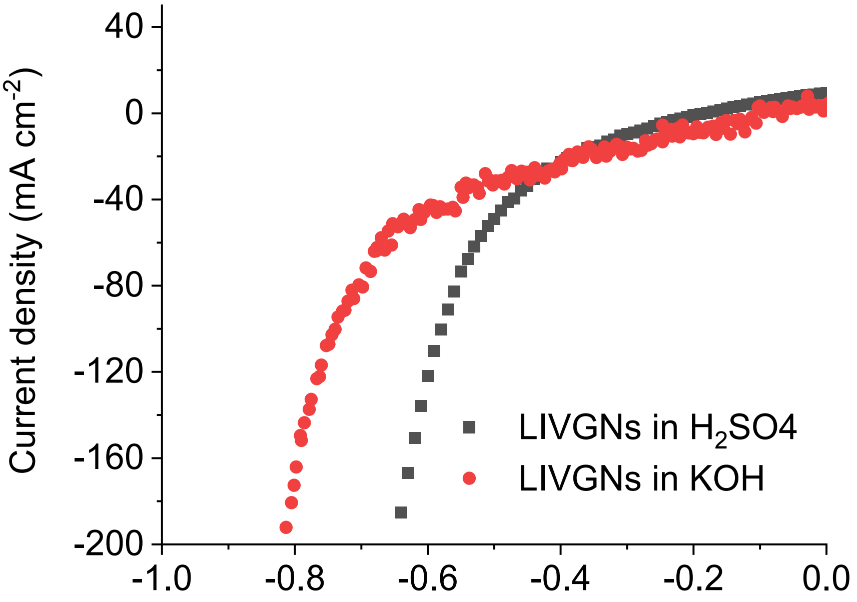

Figure S3: Comparison of LSV curves in acid (black graph) and alkaline (red graph) conditions.

Figure S4: a) O1s XPS graph of the LVGNs electrode in pristine state (a, black graph) and after being tested in long chronoamperometry test (a, red graph). b) Potassium 2s region of the same samples.

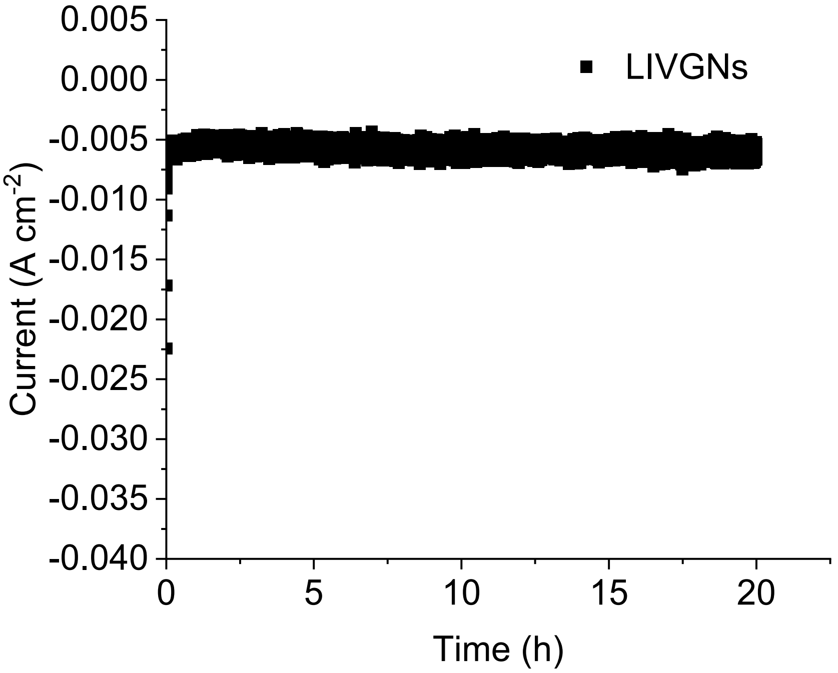

Figure S5: Long term stability of the catalyst during a 20 h- long chronoamperometry test in alkaline conditions.
